# Assessment of women’s satisfaction with family planning service at public health facilities in Northwest Region of Ethiopia: a cross sectional study

**DOI:** 10.1186/s40834-018-0079-4

**Published:** 2018-12-06

**Authors:** Woineshet Asrat, Teferi Mekonnen, Melkamu Bedimo

**Affiliations:** 1 Amhara Regional Health Office, Bahir Dar, Ethiopia; 2 Public Health Nutrition department, School of Public Health, Bahir Dar University, P.O.Box 79, Bahir Dar, Ethiopia; 3 Department of Epidemiology and Biostatistics’, School of Public Health, Bahir Dar University, Bahir Dar, Ethiopia

**Keywords:** Family planning, Client satisfaction, Public health facility, Bahir Dar Ethiopia

## Abstract

**Background:**

Ethiopia is a Sub Saharan African country with an estimated contraceptive prevalence rate of 36% and 22% unmet need for family planning service among married women. Client satisfaction influences the use of Family Planning and other reproductive health services. There is limited information on satisfaction with family planning service among Family planning users particularly in the northern part of Ethiopia. Hence, this study aimed to provide information on client satisfaction and its determinant among women in Public Health facilities of Northwestern Ethiopia.

**Methods:**

A Facility based cross-sectional study was conducted from March 1, 2017, to March 30, 2017. An exit interview using structured pretested questionnaire was conducted on randomly selected 490 women attending family planning service in Bahir Dar city, Public Health facilities of Northwest, Ethiopia. The data was cleaned, coded and entered into Epi info™ 7 statistical software and then exported and analyzed using SPSS Version 20 statistical software. A multivariable binary logistic regression model was fitted to identify factors associated with Client satisfaction. Adjusted Odds Ratio (AOR) with the corresponding 95% Confidence Interval (CI) was calculated to show the strength of association.

**Results:**

A total of 490 family planning service users were approached for an interview and making a response rate of 99.8%. The overall client satisfaction with the family planning services was found to be 66.1%. Clients who were merchants were more likely to be satisfied with the family planning service than government employees [AOR = 2.5, *P*-value = 0.007). In addition, house wife’s more likely to be satisfied with the family planning service than government employees AOR = 2.4, *P*-value = 0.007). Daily laborers were also found to be more likely to be satisfied with the FP service as compared with governmental employees AOR = 3.9, *P*-value = 0.007). New Family Planning user clients were more likely to be satisfied with the family planning service than repeat users [AOR = 2.3, *P*-value = 0.004).

Family planning service waiting time also affects client satisfaction, in which those having awaiting time of less than half an hour’s (AOR = 9.7, (*P*-value =001), One to half an hour (AOR = 6.4, (P-value =001), One hour to two hours (AOR = 4.6, (*P*-value =001) were more likely to be satisfied with the family planning service delivered at the facility than those who had waited for more than two hours. In addition, those whose privacy was maintained during counseling were more likely to be satisfied with the family planning service delivered than whose privacy was not maintained (AOR = 3.2, *P*-Value = < 0.001). Those having convenient service hour were more likely to be satisfied with the family planning service delivered than those who don’t have convenient service hour (AOR = 2.4, *p*-value = 0.002).

**Conclusion:**

The finding of this study concludes that nearly two -third of the clients were satisfied with the family planning service delivered at Public Health facilities of Northwest of Ethiopia. New family planning service users, waiting time for the service, Maintaining privacy during counseling, having convenient service hour and occupational status of the clients were the predictors for client satisfaction with family planning service delivered at Public Health facilities in Bahir Dar city.

## Background

In developing countries, maternal mortality is a major concern [1]. According to the 2015 United Nations report, around 303,000 maternal deaths were reported globally with a maternal mortality ratio of 216 per 100,000 live births [2]. Although it is noted that there is 43% decrease in maternal mortality from 1990, the death rate is still high. In Ethiopia, according to 2016 Ethiopian Demographic Health Survey report 412 pregnancy -related deaths per 100,000 live births were occurred [3]. Previous evidences revealed that family planning service utilization positively contributes to the reduction of maternal deaths. The World Health Organization (WHO) defines family planning as something that “allows individuals and couples to anticipate and attain their desired number of children and the spacing and timing of their births which can be improved by family planning counseling which deals about continuous process that the counselor as the counselor provide to help clients and people to make and arrive at informed choices about the size of their family” [4, 5]. According to lancet report, use of contraception prevents nearly 32% of maternal deaths and 10% of infant deaths [6]. After recognition of satisfaction of sexual and reproductive rights as a human rights in 1994, global efforts were made on recognition of the importance of family planning for maternal and child survival [7].

Despite these efforts, the use of modern methods as measured by the contraceptive prevalence rate (CPR) remains low in many developing countries, with a growth of 1% per year, over the last 30 years [8]. In sub-Saharan African countries the lowest contraception and highest unmet need were observed due to a mainly due to low level of knowledge and existence of variety of barriers [9, 10].

In Ethiopia, the contraceptive prevalence rate is 36% and 22% of married women have unmet need for family planning which is affected by education level, Partner education, religion, household wealth status, number of living children and media exposure [3, 11, 12]. Family planning service utilization is also affected by client satisfaction [13]. According to different studies, client satisfaction might be affected by the service waiting time [14–17], age, maternal education, cleanliness of the facility, frequency of visit, proper and adequate explanation on how to use contraceptive were factors affecting client satisfaction [18–21].

In order to improve family planning service utilization, it is better to assess levels of client satisfaction with the service delivered and its determinants. There is limited information on levels of client satisfaction with the family planning service delivered at Public Health facilities in Bahir Dar City, Northwest Ethiopia. Hence, this study will provide information on the levels of client satisfaction and its determinants delivered at public health facilities which will be used as an input for improving family planning service delivery in Northwestern part of Ethiopia.

## Methods

### Study area

This study was conducted in Bahir Dar City which is located 565 km northwest of Addis Ababa, the capital city of Ethiopia. According to Planning and Economy Bureau of Amhara Regional State report in 2016, the total population of Bahir Dar city administration was 308,877 of which 245,770 were resided in Bahir Dar town, while 63,107 are living in rural part. Out of the total population 158,824 females are eligible for family planning service use. According to 2016 zonal health department report, of the total family planning service user 55% were used family planning service from Public Health facilities.

### Study design, period, source population and study population

A Facility based cross-sectional study was conducted from March 1, 2017, to 30, 2017.. All women who were using family planning service in Bahir Dar city administration were the source population. Women’s who were using family planning service at Public Health Facility at Bahir Dar City were the study population. All women who were on family Planning service during the data collection period were included in the study.

The dependent variable was client satisfaction with family planning service delivered (“satisfied” and “Not satisfied”) and the independent variables were service waiting time, age, maternal educational status, cleanliness of the facility, frequency of visit, proper and adequate explanation on how to use contraceptive, occupational status, frequency of family planning visit, having convenient service hour, privacy during counseling were the independent variables assessed in this study.

According to this research, a client will be satisfied, if client had a mean satisfaction score of ≥3.22 which is used as a cut of to declare as a client is satisfied with the family planning service delivered. This categorization was made by dichotomizing clients satisfaction based on a mean satisfaction score after data collection during the analysis stage.

### Sample size determination and sampling procedure

The sample size was determined using Epi info™ 7 after considering the following assumptions; taking 75.3% proportion of satisfaction obtained from previous studies [18], 95% confidence limit and 4% margin of error (d) and an expected non-response rate of 10%. Finally, 491 women’s who were using family planning service were included in the study.

### Sampling procedure

A systematic random sampling technique was used for selection of the clients. The proportional allocation was made for 12 Health facilities providing family planning service at Bahir Dar city. Finally, every third family planning users were included in the study.

### Data collection technique, tools, and procedures

The Data collection was done using validated and structured pretested questionnaire through face-face interview of women at the exit of the family planning service department by trained Female data collectors. In this study***,*** the satisfaction of clients with the Family Planning service was assessed using fourteen Likert scaled question items. Each item of the question had 5 points ranging from 1(very unsatisfied) to 5 (very satisfied) and finally, the mean score was computed.

### Data quality control

Two- day training was given for data collectors and supervisors. The pre-test was made on 5% of the total sample and the questionnaire was developed in English and then translated into the local language, Amharic and back to English by language experts. Trained 12th Grade complete female data collector were recruited and regular supervision was made by supervisors.

### Data processing and analysis

The data was cleaned,coded and entered into Epi info™ 7 Statistical Software and then exported and analyzed by using SPSS version 20 statistical software. Descriptive statistics such as frequencies and percentages were used to describe the study population in relation to relevant variables. Normality assumptions were checked and the satisfaction score was normally distributed. Stepwise multivariable logistic regression model was used to identify predictors’ of client satisfaction. The goodness of fitness of the model was checked by Hosmer and Lemeshow assumption test (i.e *P*-Value 0.56). The association between dependent and independent variables were assessed using odds ratio with 95% confidence interval and *p*-value ≤0.05 was considered statistically significant.

### Ethical approval

Ethical approval was obtained from the Ethical review board of Bahir Dar University, College of Medicine and Health Sciences. Permission letters were obtained from Amhara regional Health office and Bahir Dar City Health Administration. Verbal consent was obtained from the study participants after explaining the study objectives and procedures. Finally, the date collection was made after they agreed and signed for an agreement to participate in the study. Their right to refuse from being participated was considered. Names of the participants were kept anonymous.

## Results

### Socio-demographic characteristics of study participants in Bahir Dar city

A total of 490 family planning service users were approached for an interview and making a response rate of 99.8%.

The mean (±SD) age of respondents was 27 (±5.2) years. One hundred seventy -seven (36%) of the respondents were within the age group of 25–29 years of age and four hundred seven (83.1%) of the respondents were married and more than three fourths (77.8%) of the respondents were urban residents. One hundred seventeen (23.9%) of the respondents were unable to read and write. With regarding occupational status of the respondents, one hundred sixty four (33.5%) were a housewife About 366 (88.8%) family planning users were orthodox Christian by religion (Table 1).

**Table 1:** Socio-Demographic characteristics of Family planning user’s at the public health facility in Bahir Dar city administration, from March 1,2017, to 30, 2017(*n* = 490)

Variable	Category	Number	Percent (%)
Age in years	15–19	37	7.6
	20–24	110	22.4
	25–29	177	36.1
	30–34	110	22.4
	35 & above	56	11.4
Residence	Rural	109	22.2
	Urban	381	77.8
Educational status	Unable to read and write	117	23.9
	Read and write only	90	18.4
	Grade 1 to 8	58	11.8
	Grade 9 to 12	117	23.2
	Certificate and above	108	22.7
Occupational Status	Government employee	96	19.6
	Private employee	58	11.8
	Merchant	67	13.7
	Housewife	164	33.5
	Unemployed	17	3.5
	Student	23	4.7
	Daily laborer	65	13.3
Religion	Orthodox Christian	394	80.4
	Muslim	68	13.9
	Other	28	5.7
Marital status	Married	407	83.1
	Single	62	12.7
	Divorced	17	3.5
	Widowed	4	0.8

### Family planning service- related characteristics among family planning service users in Bahir Dar city administration public health facility, 2017

Out of 490 respondents, 389(79.4%) respondents’ had received the service from the health center and chi squire test of association was done there no more significant association between hospitals and health centers in our setup (*P* = 0.19). Three hundred sixty eight (76%) of the respondents were repeat users and about three hundred three (61.8%) of participants had used Injectables. Three hundred forty three (70.0%) of the family service users had at least one child and 68.2% of respondents had received their method of choice.

Majority 448(91.4%) of respondents have to walk less than 1 h walking distance to get the service, 302(61%) of respondents reported that, the waiting time to receive the service was short and 407(83.1%) of respondents reported the service hour was convenient for them to use family planning service (Table 2).

**Table 2:** Family planning service utilization, health facility related characteristics’ among family planning user’s at public health facility in Bahir Dar city administration, from March 1,2017 to March 30, 2017(*n* = 490)

Variable	Category	Number	Percent
Types of Health facility visited	Health Center	389	79.4
	Hospital	101	20.6
Frequency of visit	New	122	24.9
	Repeat	368	75.1
Number of children	Have no child	122	24.9
	1–4 children	343	70.0
	> 4 children	25	5.1
Clients receiving their method of choice	Yes	334	68.2
	No	156	31.8
Method that the client was using	Pills	32	6.5
	Inject able	303	61.8
	Implant	126	25.7
	IUCD	29	5.9
Time it takes to reach the health facility for FP service	Less than half hour	314	64.1
	Half hour to 1 h	134	27.3
	1 to 2 h	24	4.9
	More than 2 h	18	3.7
Waiting time to received the service	Less than half hour	226	46.1
	Half hour to 1 h	210	42.9
	1 to 2 h	33	6.7
	More than 2 h	21	4.3
Perceived waiting time for the FP service	Short waiting time	302	61.6
	Long waiting time	61	38.4
FP service hour convenient for you	Convenient	407	83.1%
	Inconvenient	83	16.9%

### Client-provider interaction and information given by family planning service provider characteristics among family planning service users at public health facility in Bahir Dar city,2017

**Fig. 1:**
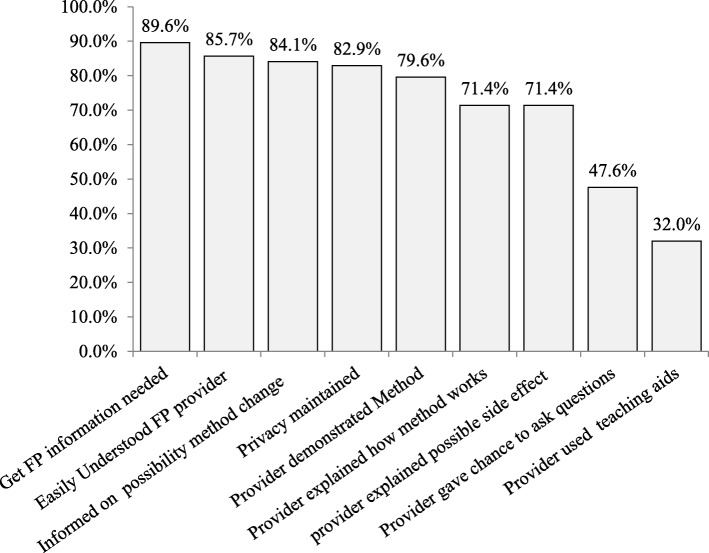
Client-provider interaction and information given by family planning service provider characteristics of family planning user’s at public health facility in Bahir Dar city administration, from March 1 to 30, 2017(n=490)

Majority of respondents, 439(89.6%) reported that, they can get information when they need about family planning from their service provider. About 420 (85.7%) of respondents could understand the family planning service provider easily during their contact time. About 233(47.6%) of the family planning user reported that, the family planning service provider were given a chance to ask questions and 256(32%) of respondents had received family planning service information through teaching aids (Fig. 1).

### Family planning service satisfaction among family planning user’s at the public health facilities in Bahir Dar city, 2017

The overall client satisfaction with the family planning services was found to be 66.1% (95% CI: 61.2%, 70.1%). Majority of the respondents (89%) reported as they were satisfied and very satisfied with the choice of method availability, 88% respondents were satisfied and very satisfied with the cleanness of procedures, and 87% were satisfied and very satisfied with the service provider knowledge and skill (Table 3).

**Table 3:** Proportion of client Satisfaction among family planning users at public health facility at public health facility in Bahir Dar city, from March, 2017(*n* = 490)

Characteristics	Very dissatisfied [No (%)]	Dissatisfied [No(%)]	Neutral [No(%)]	Satisfied [No. (%)]	Very satisfied [No. (%)]
Registration staff warmly welcomed you	31(6.3)	126(25.7)	152(31.0)	128(26.1)	53(10.8)
Professionals* informed you where FP service department	10(2.0)	102(20.8)	171(34.9)	152(31.0)	55(11.2)
Professionals* were available when required	10(2.0)	39(8.0)	126(25.7)	241(49.2)	74(15.1)
Professionals* introduce their name to you	181(36.9)	182(37.1)	57(11.6)	52(10.6)	18(3.7)
Professionals* spent enough time in consultation	19(3.9)	44(9.0)	177(36.1)	203(41.4)	47(9.6)
Professionals* were respectful	9(1.8)	67(13.7)	192(39.2)	166(33.9)	56(11.4)
Professionals* performs the procedure with cleanliness and sanitation	5(1.0)	4(0.8)	50(10.2)	287(58.6)	144(29.4)
Professionals* explanation was clear and straightforward	3(0.6)	11(2.2)	51(10.4)	266(54.3)	159(32.4)
Choice of methods available	14(2.9)	6(1.2)	32(6.5)	219(44.7)	219(44.7)
Professionals* gave adequate information	16(3.3)	43(8.8)	123(25.1)	206(42.0)	102(20.8)
Health facility easily accessible	13(2.7)	23(4.7)	119(24.3)	237(48.4)	98(20.0)
Location of family planning service department	7(1.4)	37(7.6)	160(32.7)	198(40.4)	88(18.0)
Waiting room has enough sitting chairs	33(6.7)	27(5.5)	115(23.5)	180(36.7)	135(27.6)
Cleanliness of the health facility	5(1.0)	18(3.7)	128(26.1)	228(46.5)	111(22.7)

* Professionals* in this table indicated that those who provide family planning service in Public Health facilities of Bahir Dar city administration

### Factors associated with client satisfaction among family planning service users at the public health facilities in Bahir Dar city administration, 2017

The variable such as, occupational, type of Family Planning visit, service hour convenient, privacy during counseling and waiting time for receiving the service were significantly associated with client satisfaction by using stepwise multivariable logistic regression.

Clients who were merchants were 2.5 times more likely to be satisfied with Family planning service delivered at Public Health facilities than government employees [AOR = 2.5(95% CI =1.2, 5.2)].

Clients those who are housewife were 2.4 times more likely to be satisfied with FP service delivered than government employees AOR = 2.4 (95% CI = 1.3, 4.4). Daily laborers were also found to be 3.9 times more likely to be satisfied with the FP service as compared with governmental employees AOR = 3.9(95% CI =1.8,8.6).

New family planning user clients were 2.3 times more likely to be satisfied with FP service than repeat users [AOR = 2.3 (95% CI =1.3–4.0)] (Table 4).

**Table 4:** Factors associated with client satisfaction with family planning service among respondents at public health facilities in Bahir Dar city administration, North West Ethiopia, March, 2017 (*n* = 490)

Variables		Family planning Service User		COR(95% CI)	AOR (95% CI)	*p*-value
		Satisfied (%)	Not satisfied (%)			
Marital status	Married	275(67.6%)	132(32.4%)	1.4(0.9, 2.3)*	1.76(0.9,3.3)	0.080
	Others	49(59%)	34(41%)	1	1	
Occupational status	Government employee	46(47.9%)	50(52.1%)	1	1	0.007
	Private employee	29(50.0%)	29(50.0%)	1.1(0.7,2.1)	1.2(0.6,2.5)	
	Merchant	48(71.6%)	19(28.4%)	2.7(1.4,5.3)**	2.5(1.2, 5.2)**	
	Housewife	21(73.8%)	43(26.2%)	3.1(1.8,5.2)**	2.4(1.3, 4.4)***	
	Unemployed	13(76.5%)	4(23.5%)	3.5(1.1,11.6)**	3.3(0.8,12.8)	
	Student	15(65.2%)	8(34.8%)	2.0(0.8,5.3	1.9(0.7, 5.8)	
	Daily laborer	52(80.0%)	13(20.0%)	4.3(2.1, 9.0)	3.9(1.8,8.6)***	
FP visit	New	88(72.1%)	34(27.9%)	1.2(0.9, 2.3)*	2.3(1.3,4.0)***	0.004
	Repeat	236(64.1%)	132(35.9%)	1	1	
Service hour convenience	No	35(42.2%)	48(57.8%)	1	1	0.002
	Yes	289(71.0%)	18(29.0%)	3.4(2.1,5.5)	2.4(1.4, 4.3)***	
Waiting time	< 1/2 h	166(73.5%)	60(26.5%)	6.9(2.6,18.7)**	9.7(3.2, 29.3)***	.001
	≥1/2to 1 h	132(62.9%)	78(37.1%)	4.23(1.6,11.2)	6.4(2.1, 19.2)***	
	> 1 to 2 h	20(60.6%)	13(39.4%)	3.85(1.2,12.5)	4.6(1.3, 16.7)**	
	> 2 h	6(28.6%)	5(71.4%)	1	1	
Privacy	No	33(39.3%)	51(60.7%)	1	1	< 0.001
	Yes	291(71.7%)	115(28.3%)	3.9(2.4,6.37)***	3.2(1.8, 5.5)***	

NB: *P-Value < 0.2, **P-Value < 0.05, ***p-value < 0.01

## Discussion

The result of this study revealed that 66.1% (95% CI: 61.2%, 70.1%) which is nearly two -third of respondents were satisfied with family planning services rendered by public health facilities in Bahir Dar city administration.

The overall satisfaction is low when it is compared with studies done in Jimma, south -west Ethiopia which was 77% [14] and the study done in Mozambique 86% [22]. The discrepancy might be due to the fact that the study which was conducted in Jimma, south-west Ethiopia was conducted in a specialized teaching hospital which has a relatively adequate number of health professionals and better diagnostic facilities. But for the study done in Mozambique, there is a difference in socio-demographic characteristics’ of the study participants.

In this study, occupational status was found to be the factor which affects family planning service satisfaction in which being merchant, housewife, and, daily laborer were more likely to be satisfied with the family planning service as compared to the government employee. Similar results have been reported by other authors [22–24]. The possible reason might be in our study area, the governmental working hour and family planning service hour was the same and an overlapping of this might lead to governmental employees to be dissatisfied with the family planning service.

Service hour for family planning service was also found to be one of the factors which affects client satisfaction with family planning service, in which those having convince service hour were more likely to be satisfied with family planning service as compared to those who don’t have convenient service hours. The possible reason might be due to the fact that having convenient service hour will help the women to utilize the service appropriately without overlapping of family planning service delivery and office work hour. This evidence is supported by studies done before [25].

A woman who comes for family planning service for the first time were more likely to be satisfied with the family planning service rendered by public facilities in Bahir Dar city than who had two and more family planning visits. This might be due to the fact that the doses of service given for first family planning service users is more comprehensive than those clients who are frequently using on it.

In this study, Service waiting time was also found to be the factors which affects the family planning service satisfaction in which clients having short waiting time were more likely to be satisfied as compared to those having long waiting time and this evidence was supported by other studies [26–28]. The possible reason might be, due to the fact that in the study area, the working hour is specific due to limited number of family planning serves providers [29], which might cause the service users to wait for long time.

Clients whose privacy was maintained during family planning counseling and procedures were more likely to be satisfied with the family planning service than those whose privacy was not maintained. The possible reason might be family planning is a very personal subject and people do not like to openly discuss their problems. Therefore, privacy is very much important in providing family planning services clients feel more comfortable if providers respect their privacy during counseling sessions, examinations, This evidence was supported by previous studies [18, 30].

### Limitations of the study

Since the study was cross- sectional study it is not far from pitfalls of cross -sectional study. We didn’t found comparable findings to compare our findings for discussion and we are forced to compare with studies which done in the specialized hospitals. We have used a mean satisfaction score which is not far from the limitations of using mean. There might be courtesy bias too.

## Conclusions and recommendation

The finding of this study concludes that only nearly two -third of the clients are satisfied with the family planning service delivered at Public Health facilities of Northwest Ethiopia. Frequency of visit, waiting time, privacy during counseling, having convenience of service hour and occupation of the clients were the predictors of client satisfaction with family planning service.

Hence, in order to improve client satisfaction with family planning service in northwestern Ethiopia, it is better to give attention to repeat family planning service visitor, for those government employees, maintaining privacy during counseling to family planning service, shortened service waiting time and facilitating convenience service hour for family planning service delivery. Finally, Researchers are recommended to assess determinants of family planning service satisfaction using a strong study design.
